# PVDF Fibers Modification by Nitrate Salts Doping

**DOI:** 10.3390/polym13152439

**Published:** 2021-07-24

**Authors:** Dinara Sobola, Pavel Kaspar, Klára Částková, Rashid Dallaev, Nikola Papež, Petr Sedlák, Tomáš Trčka, Farid Orudzhev, Jaroslav Kaštyl, Adam Weiser, Alexandr Knápek, Vladimír Holcman

**Affiliations:** 1 Academy of Sciences ČR, Institute of Physics of Materials, Žižkova 22, 616 62 Brno, Czech Republic; sobola@ipm.cz (D.S.); aweiser@ipm.cz (A.W.); 2 Department of Physics, Faculty of Electrical Engineering and Communication, Brno University of Technology, Technická 2848/8, 616 00 Brno, Czech Republic; kasparp@feec.vutbr.cz (P.K.); rashid.dallaev@vutbr.cz (R.D.); Nikola.Papez@vutbr.cz (N.P.); sedlakp@feec.vutbr.cz (P.S.); holcman@feec.vutbr.cz (V.H.); 3 Department of Inorganic Chemistry and Chemical Ecology, Dagestan State University, St. M. Gadjieva 43-a, 367015 Makhachkala, Russia; farid-stkha@mail.ru; 4 Central European Institute of Technology BUT, Purkyňova 123, 612 00 Brno, Czech Republic; klara.castkova@ceitec.vutbr.cz (K.Č.); jaroslav.kastyl@ceitec.vutbr.cz (J.K.); 5 Department of Ceramics and Polymers, Faculty of Mechanical Engineering, Brno University of Technology, Technická 2, 616 69 Brno, Czech Republic; 6 Institute of Scientific Instruments of the Czech Academy of Sciences, Královopolská 147, 612 64 Brno, Czech Republic; knapek@isibrno.cz

**Keywords:** PVDF, nitrate salt, XPS, XRD, Raman spectroscopy, FTIR, EDX, permittivity

## Abstract

The method of inclusion of various additives into a polymer depends highly on the material in question and the desired effect. In the case of this paper, nitride salts were introduced into polyvinylidene fluoride fibers prepared by electrospinning. The resulting changes in the structural, chemical and electrical properties of the samples were observed and compared using SEM-EDX, DSC, XPS, FTIR, Raman spectroscopy and electrical measurements. The observed results displayed a grouping of parameters by electronegativity and possibly the molecular mass of the additive salts. We virtually demonstrated elimination of the presence of the γ-phase by addition of Mg(NO_3_)_2_, Ca(NO_3_)_2_, and Zn(NO_3_)_2_ salts. The trend of electrical properties to follow the electronegativity of the nitrate salt cation is demonstrated. The performed measurements of nitrate salt inclusions into PVDF offer a new insight into effects of previously unstudied structures of PVDF composites, opening new potential possibilities of crystalline phase control of the composite and use in further research and component design.

## 1. Introduction

Composite polymer materials have attracted a lot of attention from the scientific community. One of the most prominent polymers in question is polyvinylidene fluoride (PVDF), because of its high chemical and physical resistance and biocompatibility. PVDF thin films and their properties have been explored by a number of authors [1,2,3]. The fibrous form of the polymer carries different properties and possibilities. That said, special care must be taken in their manufacture, because the preparation process of fibers can be difficult to achieve safely and reliably [4]. The process that is most commonly used in the present to create suitable fibers is electrospinning, providing the best control over the parameters of the resulting material, with diameter of the fiber, inclusions and crystalline phases being among them [5,6]. Many properties of PVDF stem from the concentration of crystalline phases and as such they are one of the most important topics of conversation when the structural, chemical and electrical properties of these fibers are concerned [7,8].

Even though it is possible to affect the composition phase-wise just by proper selection of electrospinning properties, there is a limit to how much control can be exerted over the phase formation process. To surpass that limit and to change the related properties of the fibers even further, other materials can be included into the polymer during the manufacture [9,10,11].

The novelty of this work lies with the thorough investigation of the structural properties and behavior of polyvinylidene fluoride electrospun fibers with nitrate salt inclusions. In this paper, the focus is on nitrate salts as an additive. While their introduction into polymers is not a new topic [12], and there has been some experimentation with electrospun fibers enhanced with salts [13], the overall effect of nitrate salts on PVDF fibers has not been explored yet.

The content of the present work includes PVDF fibers preparation by electrospinning and the samples characterization by several complementary techniques, based on different physical principles in order to obtain reliable data. The research was conducted with an emphasis on the comparison of and PVDF fibers with Ca(NO_3_)_2_, Mg(NO_3_)_2_ and Zn(NO_3_)_2_ salts.

## 2. Materials and Methods

The polyvinylidene fluoride (Sigma Aldrich, St. Louis, MO, USA) material being used in the measurements described below was prepared as fibers (*M*w = 275,000 g × mol^−1^) by electrospinning from 15 wt % PVDF solution in a blend of dimethylsulfoxide (Sigma Aldrich, St. Louis, MO, USA) and acetone (Sigma Aldrich, St. Louis, MO, USA) with a volume ratio of 7/3. The calcium, magnesium and zinc nitrates (in their hydrated form) (Lach-Ner, Neratovice, Czech Republic) were dissolved in the blending solvent in 8 wt% to the solid polymer before the PVDF dissolution. This solution was then electrospun under a constant voltage of 50 kV into fibers forming a mat with thickness of 25 μm.

Electrospinning was performed on an equipment 4-SPIN (Contipro, Dolní Dobrouč, the Czech Republic) at a feeding rate of 20 µL × min^−1^ using a thin needle with a diameter of 1.067 mm (17 G). The rotation collector has been covered with an aluminum foil to gather the resulting fibers at a speed of 2000 rpm for 30 min, with the distance between the needle tip and the collector being kept at 20 cm. The resulting non-woven fiber mats were left to dry overnight at laboratory temperature. The diameter of the fibers created by this method was in the range from 300 to 700 nm. 

X-ray photoelectron spectroscopy (XPS) was conducted to determine types of chemical bonds of the samples on AXIS Supra device (Kratos Analytical Ltd., Manchester, UK), with the results being captured under an emission current of 15 mA and resolution of 20 for wide spectra and 80 for the element-specific spectra. The resulting spectra were fitted in the CasaXPS software (version 2.3.23, Kratos Analytical Ltd., Manchester, UK) using Gaussian-Lorentzian line shape.

FTIR data were acquired in order to analyze phase composition of the samples on a Bruker device (Billerica, MA, USA) in transmission mode over 512 iterations with a resolution of 1 cm^−1^.

X-ray powder diffraction (XRD) analysis was performed for confirmation of the crystalline structure of the samples on Rigaku SmartLab 3 kW device (Rigaku, Tokyo, Japan) in the Bragg–Brentano configuration. The resulting diffraction patterns were obtained between 10° and 25° (2θ) with Cu Kα radiation.

The Raman spectroscopy results were taken for analysis of structural features of the samples by a WITec alpha300 R (WITec, Ulm, Germany) device at an excitation wavelength of 532 nm and the laser power of 10 mW. The obtained signal was reconstructed over 20 accumulations with the integration time of 10 s.

Energy-dispersive X-ray spectroscopy (EDX) was performed to follow the homogeneity of elements distribution and was conducted at an acceleration voltage of 15 kV in order to provide an overview of the elements distribution at the surface of the fibers samples. The electron microscope used was a Tescan LYRA3 (Tescan, Brno, Czech Republic) with an X-Max50 EDS detector from Oxford Instruments.

The measurement of dielectric properties, as parameters that define samples functionality, was carried out on a Novocontrol Alpha Analyzer device (Novocontrol Technologies, Montabaur, Germany) in the frequency range of 1 to 100,000 Hz.

Data from optical spectroscopy were obtained to study transmittance of the samples on a 3-channel (200–450 nm, 400–750 nm, 700–1000 nm) Spectrometer Ocean Optics JAZ-3 (Dunedin, FI, USA) using Ocean Optics software (OceanView, Dunedin, FI, USA). The resulting data were averaged from 20 scans each over 31 ms integration period.

Differential scanning calorimetry (DSC) measurements were performed to define crystallinity on DSC 204 F1 (NETZSCH, Selb, Germany) at a heating rate of 10 °C × min^−1^ from 25 °C to 200 °C under an argon flux 20 mL × min^−1^.

Every measurement was performed five times for every sample for good data reliability. With the exception of DSC, all of the measurements used were carried out under room temperature.

## 3. Results and Discussion

**Figure 1:**
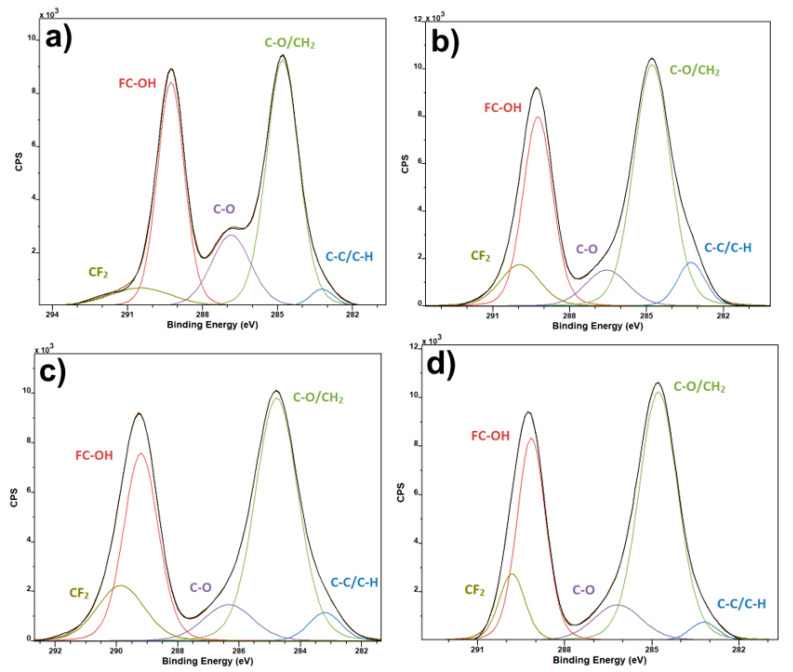
XPS C1 spectra of pure PVDF fibers (**a**) and PVDF fibers with Ca(NO_3_)_2_ (**b**), Mg(NO_3_)_2_ (**c**) and Zn(NO_3_)_2_ (**d**).

**Figure 2:**
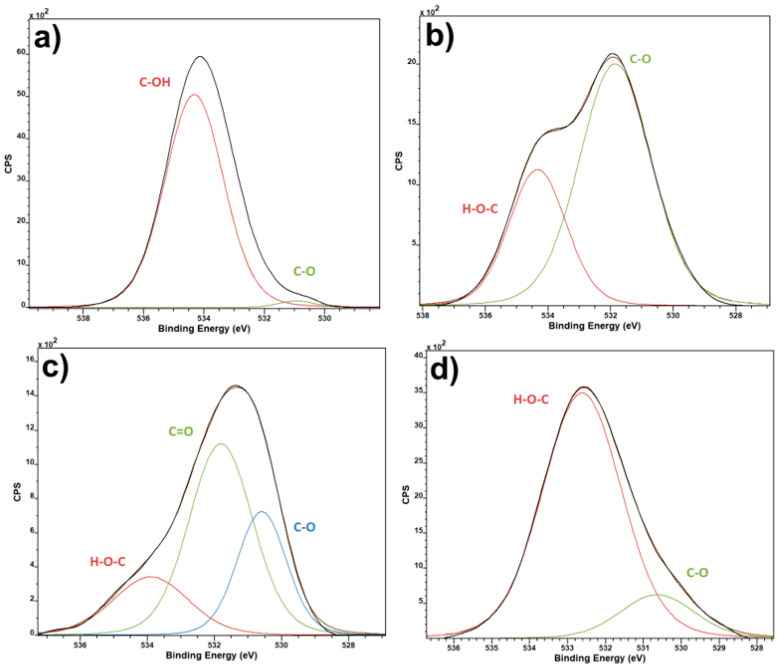
XPS O1 spectra of pure PVDF fibers (**a**) and PVDF fibers with Ca(NO_3_)_2_ (**b**), Mg(NO_3_)_2_ (**c**) and Zn(NO_3_)_2_ (**d**).

Results from the C1 spectra of XPS measurement of the materials show the presence of standard bands expected for PVDF (Figure 1) [14]. In the pure and the enhanced PVDF fibers the most prominent peak is the C–O/CH_2_ and this peak stays almost the same in all the materials. The FC–OH and C–O bands are greater in the pure PVDF, and smaller in the three doped materials. CF_2_ is the smallest in the pure material, but together with C–C/C–H they differ within each of the sample. According to literature [15], the points of deliquescence are 56% for Mg(NO_3_)_2_·6H_2_O and Ca(NO_3_)_2_·4H_2_O, 42% for Zn(NO_3_)_2_·6H_2_O. It explains dominant amount of H–O–C bonds for PVDF fibers with Zn(NO_3_)_2_ (Figure 2b).

O1 spectra (Figure 2) of pure PVDF samples and salt-enhanced ones show one interesting fact, that the PVDF with calcium and magnesium salts have much higher relative content of C-O bonds, than zinc salt and pure PVDF. This points to presence of CO_2_ in the materials, as there seems to be no other reason for such an increase in the C–O bonds. The only time that such a contamination could occur is during the electrospinning process due to high reactivity of the two elements. The CO_2_ presence is not intended, and the salts in the composite are much more reactive than CO_2_, being the reason for its presence in the samples. The salts have an effect on the chemical bonds and crystalline composition of the resulting fiber. As it will be shown below, the crystallinity of PVDF with calcium and magnesium salts is lower, which make possible CO_2_ capture [16].

**Figure 3:**
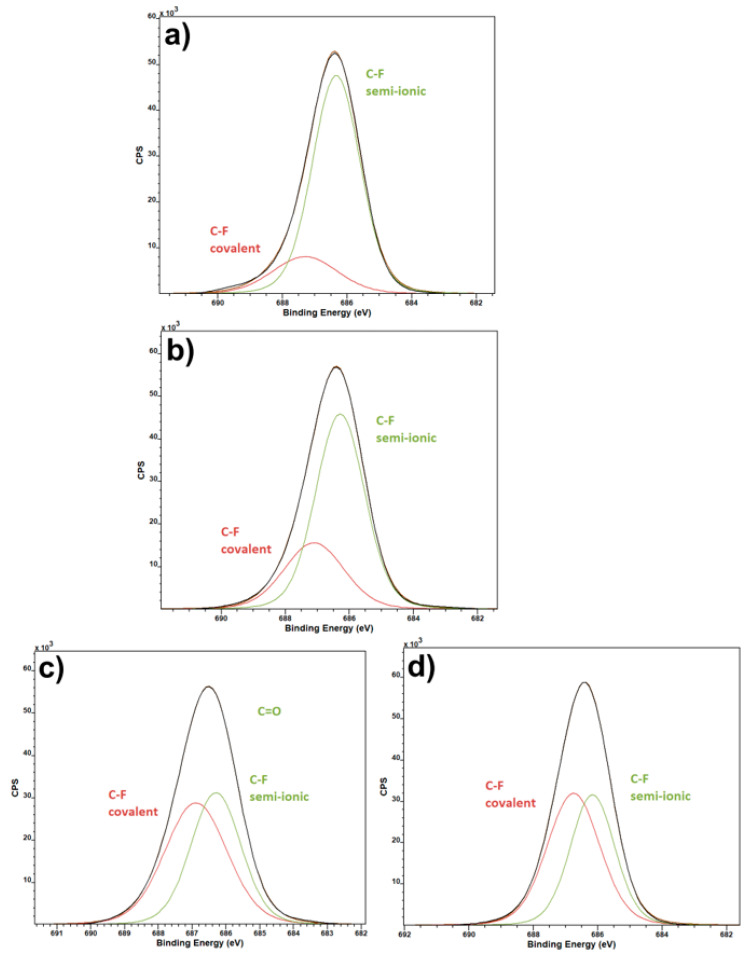
XPS F1s spectra of pure PVDF fibers (**a**) and PVDF fibers with Ca(NO_3_)_2_ (**b**), Mg(NO_3_)_2_ (**c**) and Zn(NO_3_)_2_ (**d**).

F1 spectra (Figure 3) are interesting namely because of the indication of covalent and semi-ionic bond presence. Pure PVDF has a high ratio of semi-ionic to covalent bonds. This does not change much in the calcium salt variant, but the ratio is almost even in the magnesium and zinc salt variants. This can be likely ascribed to the electronegativity of the elements in question. Calcium electronegativity is lower compared to the other two salt elements, which causes the ratio of covalent and semi-ionic bonds to remain almost the same as pure PVDF, when compared to the other two salts.

**Figure 4:**
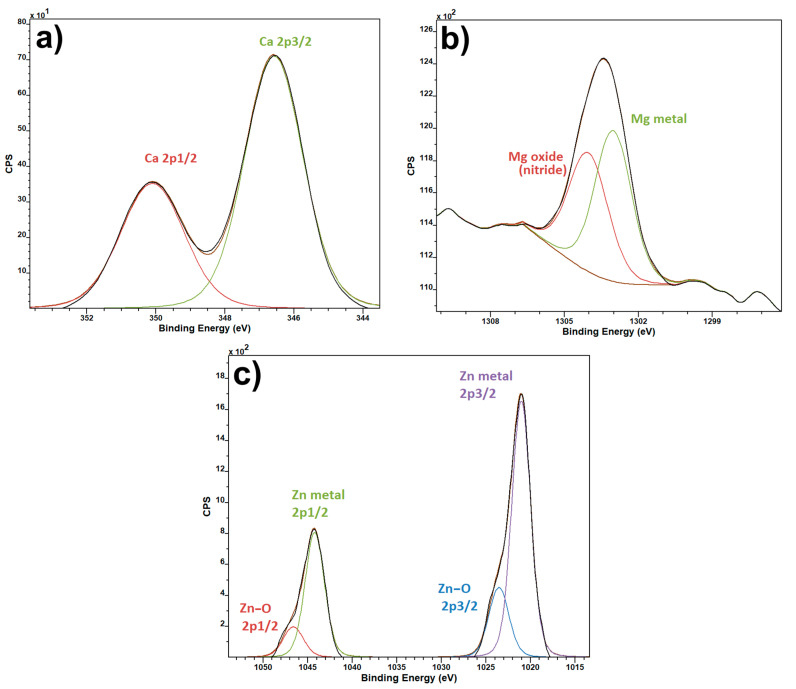
XPS spectra of unique salt elements: Ca2p for PVDF fibers with Ca(NO_3_)_2_ (**a**), Mg1s for PVDF fibers with Mg(NO_3_)_2_ (**b**) and Zn2p for PVDF fibers with Zn(NO_3_)_2_ (**c**).

Unique elements spectra (Figure 4) serve as a check for the presence of the specified salts in the intended samples. Since it is virtually impossible to tell whether or not the salts are present by eye, or even by scanning electron microscopy, XPS spectra of the unique elements were used as a confirmation that the required elements have made their way from the electrospinning solution into the final fiber product. 

**Figure 5:**
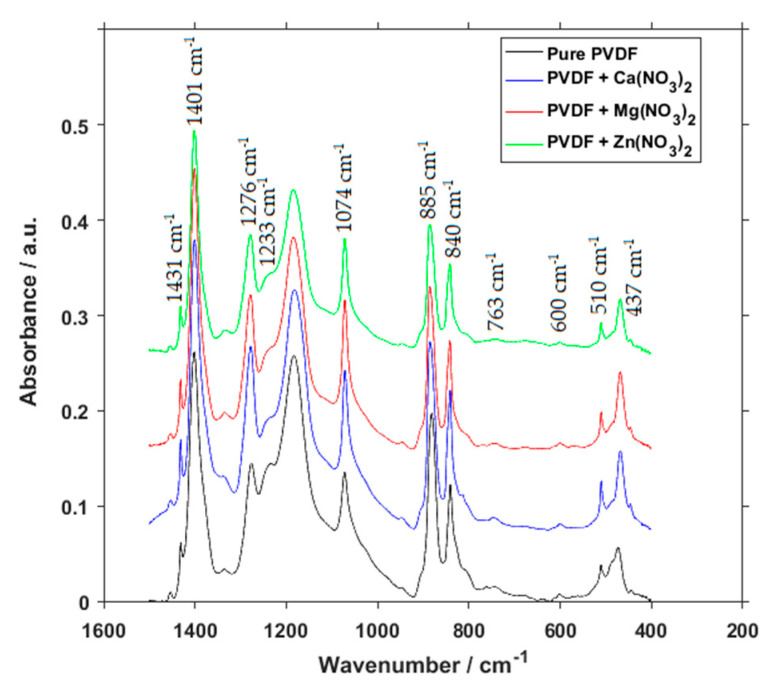
FTIR spectra of pure PVDF fibers and PVDF fibers with Ca(NO_3_)_2_, Mg(NO_3_)_2_ and Zn(NO_3_)_2_.

To gain a more in-depth look into the presence and concentration of crystalline phases, FTIR measurement was performed (Figure 5). Peaks at 510 cm^−1^ represent both the β and γ phases, but as is the case for all of the peaks combining multiple phases, it is not possible to discern the concentration of individual phases from them. A small peak at 600 cm^−1^ is sometimes assigned to the β phase [17,18,19,20], but some specialized authors argue that it should not be so, as this band commonly shows up in many samples, including those with pure α phase, due to more pronounced peak at 613 nm^−1^ [21]. Peaks at 840 cm^−1^ are commonly assigned to both β and γ phases. Peaks at 885 and 1401 cm^−1^ represent all three phases combined. The peak located at 1074 cm^−1^ is usually assigned to the β phase, but it is not reliable as a characteristic peak for this crystalline phase, as references to other phases can be found at this wavelength as well. Lastly, from the combined peaks, the one located at 1074 cm^−1^ represents both the β and γ phases. Peaks characteristic for specific phases are 1233 cm^−1^ representing γ phase, and peaks located at 437, 840, 1276 and 1431 cm^−1^ represent β phase, which is the reason for their size. 

At the first glance we can see that there is only a small difference between individual spectra. If we are to look at the characteristic peaks, however, we find, that in comparison to pure PVDF, the compounds with salts have little to no characteristic peaks for γ phase. Especially the 1233 cm^−1^ most often used for determination of γ phase concentration [22] is so small that it is virtually zero. When the precision of FTIR is taken into account, the presence of γ phase can be considered to be negligible. 

Calculation of phase composition was performed according to X. Cai, et al. [21]

(1) $$F_{EA}=\frac{I_{EA}}{\left(\frac{K_{840}}{K_{763}}\right)I_{763}+I_{EA}}\cdot 100\%$$

(2) $$F\left(\beta \right)=F_{EA}\cdot \frac{\Delta H_{\beta^{\prime }}}{\Delta H_{\beta^{\prime }}+\Delta H_{\gamma^{\prime }}}\cdot 100\%$$

(3) $$F\left(\gamma \right)=F_{EA}\cdot \frac{\Delta H_{\gamma^{\prime }}}{\Delta H_{\beta^{\prime }}+\Delta H_{\gamma^{\prime }}}\cdot 100\%$$

Here F*_EA_* is the common amount of the electroactive phases (β and γ);

*I_EA_* is the absorbance at 840 cm^−1^;

*I*_763_ is the absorbance at 763 cm^−1^;

*K*_840_ is the absorption coefficient at the wave number 840 cm^−1^, equal to 7.7·10^4^ cm^2^·mol^−1^;

*K*_763_ is the absorption coefficient at the wave number 763 cm^−1^, equal to 6.1·10^4^ cm^2^·mol^−1^;

Δ*H*_β′_ is the height differences at absorbance spectra between the peak around at 1275 cm^−1^ and the valley around at 1260 cm^−1^;

Δ*H*_γ′_ is the height differences at absorbance spectra between the peak around at 1234 cm^−1^ and the nearest valley around at 1225 cm^−1^.

Calculation themselves show the relative fraction of phases for pure PVDF to be 13.45% for α, 82.52% for β and 4.03% for γ. This changes into 7.71% for α, 92.29% for β and virtually 0 for γ in the case of Ca(NO_3_)_2_, 4.25% for α, 95.75% for β and 0 for γ in the case of Mg(NO_3_)_2_, and 14.70% for α, 85.30% for β and again 0 for γ in the case of Zn(NO_3_)_2._ Recent theoretical research [23] confirmed the β phase formation due to interactions between polymer chains and the interstitial water of the hydrated salts through hydrogen bonds formation. Electrostatic forces caused repelling of negative charges of the polymer molecules, which is the reason for the stretching of the macromolecules and transformation into an all-trans-configuration.

**Figure 6:**
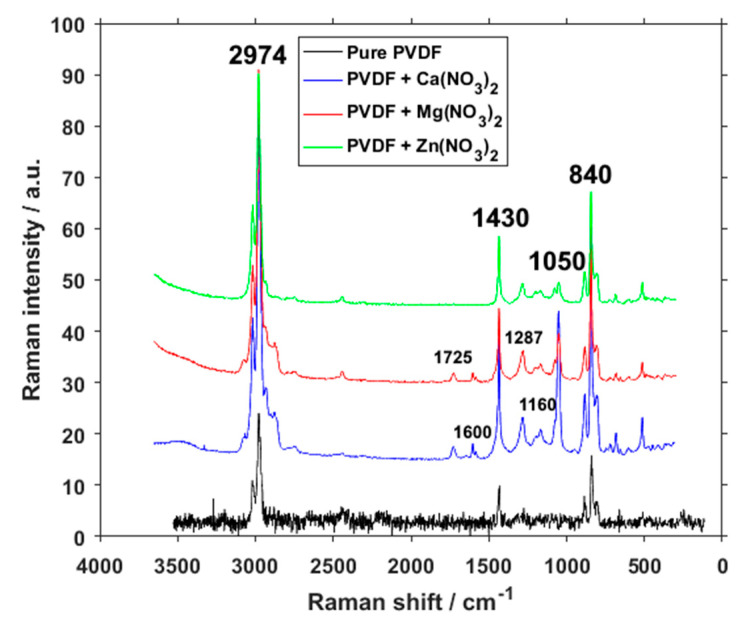
Raman spectra of pure PVDF fibers and PVDF fibers with Ca(NO_3_)_2_, Mg(NO_3_)_2_ and Zn(NO_3_)_2_.

Samples of pure PVDF and PVDF mixed with additives in the form of three different salts have been measured by Raman spectroscopy (Figure 6). From the typical expected peaks of PVDF we can find those in all four material compositions at 840 cm^−1^ belonging to β- and γ- phases, and at 1430 cm^−1^ representing the CH_2_ bending vibrations of all three phases, but which are usually attributed to the β- and γ-phases as well, and a composite band around 2974 cm^−1^ most commonly attributed to CH_2_ symmetric stretching associated with the β-phase [24]. The change in the dimensions of peaks belonging to the β-phase of PVDF, which is the one that varies the most throughout the samples, corresponds with FTIR measurements of crystalline phase concentrations.

The band at 1050 cm^−1^ that is only present in the salt-enhanced polymer belongs to a υ_1_-NO_3_^-^ group vibration [25]. Peaks at around 1170 cm^−1^ can be assigned to the β-phase [26], and since they are larger in calcium and magnesium salts, that would correspond to the concentration of phases as gained from FTIR. Peaks at around 1160 and 1287 cm^−1^ could be attributed to C–O and C=O bonds in the organic compounds [27]. A higher presence of C–O and C=O bonds can also be seen on O1 XPS spectra (Figure 2). Since the salts themselves do not contain any carbon and this peak is missing within the pure PVDF, their presence suggests either an adsorbed CO_2_ during the electrospinning process under high voltage, chemical bond between the salts and PVDF fibers, or even a possibility of PVDF hydroxylation [28]. Because these peaks are present in two different sample series, however, it is highly unlikely that they were caused by a random error or simple contamination of the source solution.

The two small peaks present at calcium and magnesium salt additives at around 1600 and 1725 cm^−1^ are bending water peaks [29], likely caused by the water absorption of these two materials, and the O–H bending peak respectively. The absence of these peaks in the spectrum of zinc salt corresponds with the water-absorption ability, which is lower in zinc salts than in magnesium and calcium ones. 

**Figure 7:**
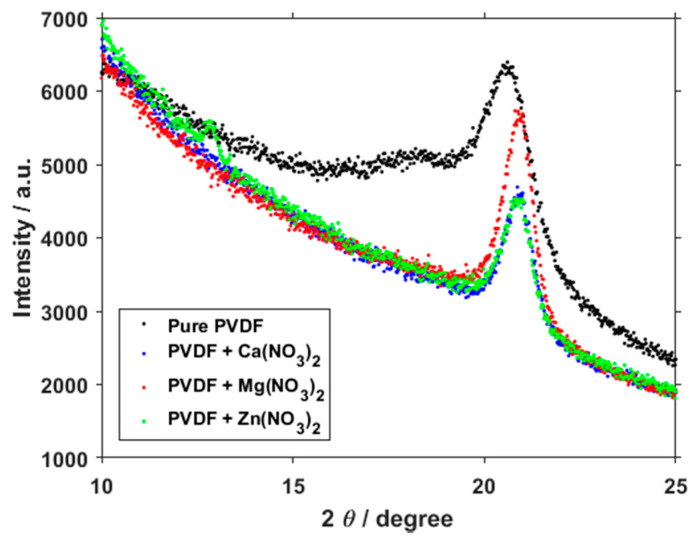
Focused XRD spectra of PVDF fibers and PVDF fibers with Ca(NO_3_)_2_, Mg(NO_3_)_2_ and Zn(NO_3_)_2_.

Several places of interest have been revealed in the spectra taken by the XRD measurement (Figure 7). The most notable is located at 18°, representing the α phase, and a second at 21°, representing a combination of the α and β phases [30]. In the pure sample, the peak at 18° is still visible, but it virtually disappears in the salt-doped PVDF fibers. This change in peaks shows a clear shift towards a very high concentration of the β phase at the expense of other phases, which corresponds with the data gained from the FTIR.

**Figure 8:**
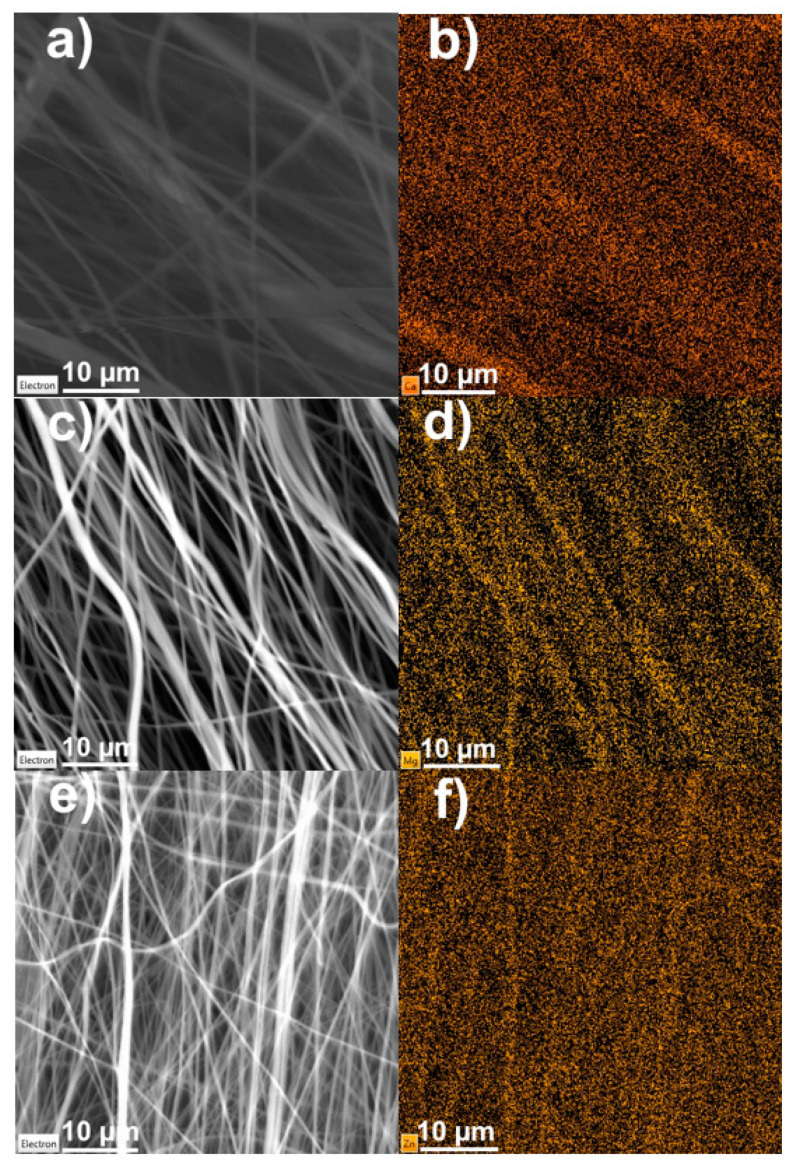
SEM and EDX images of salt-enhanced PVDF fibers, electron image and unique elements: (**a**,**b**) Ca(NO_3_)_2_, (**c**,**d**) Mg(NO_3_)_2_ and (**e**,**f**) Zn(NO_3_)_2_.

During the electrospinning process there is always the remote possibility that the inclusions will aggregate into one location and will not be distributed throughout the material. This would, naturally, affect the resulting material in a detrimental way. EDX measurement was performed to find out whether this sub-optimal distribution pattern occurred. Figure 8 shows SEM images of the samples and then EDX measurements of the same location focused on the unique element in the nitrate salts. Even though the signal-to-noise ratio is not optimal, the unique element images show the dispersion of the element in question throughout PVDF fibers, supporting the claim that this method of fiber preparation can indeed distribute the inclusions successfully and evenly.

**Figure 9:**
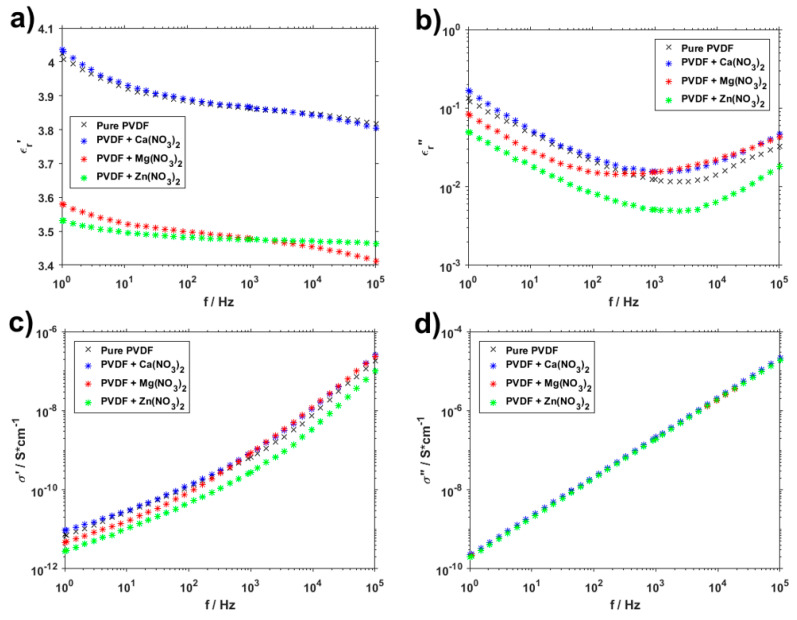
Real (**a**) and imaginary (**b**) permittivity and real (**c**) and imaginary (**d**) conductivity of pure PVDF fibers and PVDF with Ca(NO_3_)_2_, Mg(NO_3_)_2_ and Zn(NO_3_)_2_.

Figure 9 shows the dielectric measurement performed on pure PVDF samples and samples with Ca(NO_3_)_2_, Mg(NO_3_)_2_ and Zn(NO_3_)_2_. They all follow generally the same trends, but there are some differences. The first thing that is immediately visible is that the pure PVDF and PVDF with Ca(NO_3_)_2_ have highly similar progressions in the dielectric constant measurements for both real (ε_r_′) and imaginary (ε_r_″) values. This similarity is even greater in the real part of conductivity (σ′), and they are virtually the same in the imaginary part (σ″). This pairing correlates with the electronegativity of calcium and the ratio of semi-ionic and covalent bonds in Figure 3. It is originated from electrostatic interactions between the PVDF polymer chain and dissolved hydrate metallic salts caused by hydrogen bonding between the CF_2_ and the salt cation [31]. More interesting progressions can be seen in the PVDF fibers containing Mg(NO_3_)_2_ and Zn(NO_3_)_2._ The curve of the real dielectric constant for the magnesium salt intersects the one for zinc salt between 10^2^ Hz and 10^3^ Hz, which is all the more visible in the graph depicting dielectric loss. What is somewhat unexpected, however, is that conductivity of PVDF with and without added salts is almost identical, with only minor differences that would likely not be noticeable in most practical uses.

**Figure 10:**
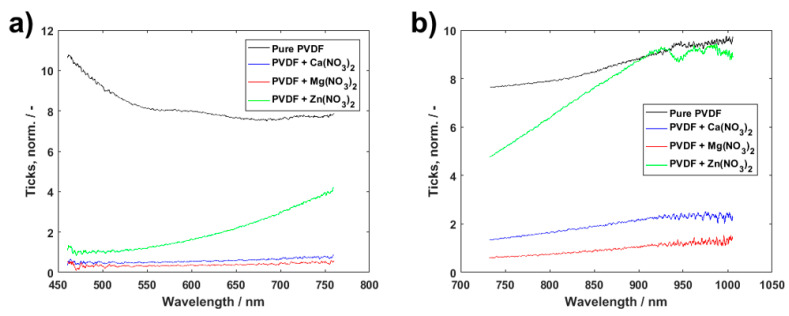
Transmittance spectra of pure PVDF fibers and those with Ca(NO_3_)_2_, Mg(NO_3_)_2_ and Zn(NO_3_)_2_ in visual (**a**) and near-infrared (**b**) range.

To obtain a more complex idea about the properties of PVDF fibers, optical spectroscopy has been performed on the materials. Though it is one of the less used methods to describe PVDF composites, it can provide more of an insight into their potential use. Pure PVDF has been used as a benchmark to compare the materials enriched by salts to. Figure 10 shows visual and near infrared transmittance spectra of the materials. While the spectra of salt-enhanced PVDF seem to have much higher absorption in lower wavelengths, zinc salt starts to be more transparent for the incident light over the course of wavelength increase, and eventually reaches the transmittance values of pure PVDF in near-infrared ranges. This makes the zinc salt optically interesting, and it might be possible to find use for the PVDF fibers enriched by Zn(NO_3_)_2_ in components actively using light for their operation.

A hydrophobicity measurement was taken to obtain information about the interaction of the material with aqueous substances, because such an interaction is both expected, and in the case of potential use in biology and chemistry, required. From the results in Table 1, it can immediately be seen that the pure PVDF material and all of its enhancements with salts are highly hydrophobic. While that is not entirely unexpected, what is more interesting is the fact that even after the addition of the salts the hydrophobicity does not change all that much. When considering the deviations, the average values of measured angles are very close to one another. As salts are usually used to alter hydrophobicity of a material [32], this lack of significant difference is somewhat puzzling. Out of all the three salts, the zinc salt behaves in the most distinctive manner, but that can be seen in the permittivity or optical spectroscopy measurement as well. 

**Figure 11:**
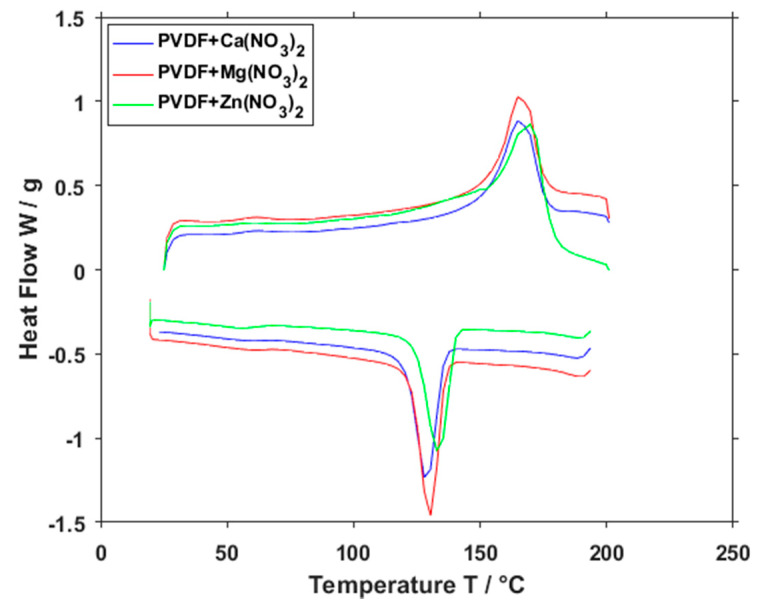
DSC curve of PVDF fibers with Ca(NO_3_)_2_, Mg(NO_3_)_2_ and Zn(NO_3_)_2_.

To obtain the crystallinity percentage of the measured material, the DSC method has been employed. Spectra gained by this evaluation (Figure 11) can be used to calculate the total sample enthalpy and then the crystalline phases (*Xc)* of the PVDF in different polymer mixtures:

(4) $$Xc=\frac{\Delta H_{f}}{\Delta H_{f}^{*}\varphi }100\%$$

where $\Delta H_{f}$ is the enthalpy of fusion, calculated from the heating DSC curve, $\Delta H_{f}^{*}$ is the heat of fusion of perfect crystalline PVDF obtained from literature [33], in this case 104.7 J/g, and φ is the weight fraction of PVDF in the samples. The obtained resulting percentage of crystallinity was 64.58% for Ca(NO_3_)_2_, 61.01% for Mg(NO_3_)_2_ and 73.46 for Zn(NO_3_)_2_. Once again, the zinc salt behavior is the most distinct out of the three measured salts. 

The hydrogen bonds play an important role in the crystallization of the polymers. These bonds could be considered as electrostatic interaction. The presence of the hydrogen atoms changes in alignment and the distance between the dipoles. The ionic interactions of the polymer with the hydrated salt as well as the hygroscopic properties of the additive are the reasons for the hydrogen bonding [34]. It effects the crystallinity which grows with the electronegativity of the nitrate salt cation. 

## 4. Conclusions

The addition of various nitrate salts has a noticeable and measurable effect on many properties of polyvinylidene fluoride fibers. One of the main observed properties was the concentration of crystalline phases. When compared to pure PVDF, salts containing calcium and magnesium cations had their β-phase content increased by a large amount, while zinc nitrate has a comparable β-phase content as pure PVDF. Any salt addition, however, virtually eliminated the presence of the γ-phase altogether. Zinc nitrate also has distinctive optical properties with a measured increased transmittance towards the higher wavelengths of the visual spectrum and further, showing a potential for components where incident light plays a key role. The measured electrical properties tend to follow the electronegativity of the nitrate salt cation, where calcium exhibits a similar ratio of covalent to semi-ionic bonds, measured by XPS, and dielectric constant, as PVDF, while magnesium and zinc form a group of their own with the dielectric constant being visibly lower. The data obtained from several different measurements have been documented to create a basis for further research and to widen knowledge about the properties of polyvinylidene fluoride fiber composites.

## Figures and Tables

**Table 1:** Average, median and standard deviations for hydrophobicity measurement of pure PVDF fibers and PVDF with Ca(NO_3_)_2_, Mg(NO_3_)_2_ and Zn(NO_3_)_2_.

	Average/°	Median/°	Std. Dev./°	Std. Dev./%
PVDF	127.1	127.6	7.6	6.0
PVDF with Ca(NO_3_)_2_	124.1	125.2	11.9	9.6
PVDF with Mg(NO_3_)_2_	131.3	131.4	4.6	3.5
PVDF with Zn(NO_3_)_2_	135.9	135.9	7.8	5.8

## Data Availability

The research data are available upon request from authors.
